# Semaglutide as a potential therapeutic alternative for HNF1B-MODY: a case study

**DOI:** 10.3389/fendo.2024.1294264

**Published:** 2024-03-08

**Authors:** Angham Almutair, Beshaier Almulhem

**Affiliations:** ^1^ Pediatric Department, King Abdullah Specialized Children’s Hospital, King Abdulaziz Medical City, Ministry of National Guard Health Affairs, Riyadh, Saudi Arabia; ^2^ College of Medicine, King Saud bin Abdul-Aziz University for Health Sciences, Ministry of National Guard Health Affairs, Riyadh, Saudi Arabia; ^3^ King Abdullah International Medical Research Center, Ministry of National Guard Health Affairs, Riyadh, Saudi Arabia; ^4^ College of Medicine, Imam Abdulrahman Bin Faisal University, Dammam, Saudi Arabia

**Keywords:** HNF1B-MODY, semaglutide, successful, MODY, GLP-1RA

## Abstract

Maturity-onset diabetes of the young (MODY) is a grouping of monogenic disorders. It is characterized by dominantly inherited, non-insulin-dependent diabetes. MODY is relatively rare, encompassing up to 3.5% in those diagnosed under 30 years of age. Specific types are most commonly treated with sulfonylurea, particularly those identified as HNF4A-MODY and HNF1A-MODY. HNF1B-MODY is another type that is most frequently managed with insulin therapy but lacks a defined precision treatment. We present an 18-year-old, non-obese female patient diagnosed with HNF1B-MODY. She displays complete gene deletion, a renal cyst, and hypomagnesemia. Her treatment plan includes both long- and short-acting insulin, though she frequently encountered hypoglycemia and hyperglycemia. Semaglutide, a GLP-1RA, was administered weekly over 4 months. The patient’s glucose level was continuously tracked using Dexcom’s Continuous Glucose Monitoring system. The data suggested a notable improvement in her condition: time-in-range (TIR) increased from 70% to 88%, with some days achieving 100%, and the frequency of hypoglycemic episodes, indicated by time-below-range values, fell from 5% to 1%. The time-above-range values also dropped from 25% to 10%, and her HbA1c levels declined from 7% to 5.6%. During the semaglutide therapy, we were able to discontinue her insulin treatment. Additionally, her body mass index (BMI) was reduced from 24.1 to 20.1 kg/m^2^. However, the semaglutide treatment was halted after 4 months due to side effects such as nausea, vomiting, and reduced appetite. Other contributing factors included exam stress and a COVID-19 infection, which forced a switch back to insulin. Her last recorded HbA1c level under exclusive insulin therapy rose to 7.1%, and her BMI increased to 24.9 kg/m^2^. In conclusion, semaglutide could potentially replace insulin to improve glucose variability, TIR, and HbA1c in patients with HNF1B-MODY. However, more extensive studies are required to confirm its long-term safety and efficacy.

## Introduction

Maturity-onset diabetes of the young (MODY) is a genetically inherited disease caused by specific mutations that affect the development of pancreatic beta cells (1). HNF1B-MODY is one of more than 40 known subtypes of monogenic diabetes, each possessing its own unique inheritance pattern and phenotype (2). There have been at least 14 MODY types identified, representing 1%–5% of all diabetes mellitus cases, with an estimated prevalence of 1–5 per 10,000 people, according to European studies (3).

The most common types of MODY are GCK-MODY and HNF1A-MODY. HNF1B-MODY, however, is not as prevalent in the UK population (4). Most people with GCK-MODY do not require treatment. HNF4A-MODY and HNF1A-MODY, in contrast, can be effectively treated with sulfonylurea. However, patients with HNF1B-MODY are typically not responsive to sulfonylurea treatment and often require insulin injections multiple times a day (5).

The HNF1B gene is essential for the embryonic development of several tissues, such as the liver, kidney, gut, and pancreatic islet cells. It also contributes to the exact regulation of gene expression in these tissues (6), explaining the varying symptoms of the associated disease. Multiple phenotypes linked to HNF1B-MODY are observed. These include cystic kidneys, unexplained renal dysfunction, genitourinary abnormalities, gout, hypomagnesemia, primary hyperparathyroidism, abnormalities in the liver and intestines, and a rare type of kidney cancer (7).

The insufficient response to sulfonylurea and the early need for insulin can be attributed to a certain level of hepatic insulin resistance and insulin deficiency resulting from pancreatic hypoplasia (8). This case report describes an 18-year-old female patient with HNF1B-MODY, who was discovered to have a complete gene deletion (heterozygous for a 1.82-Mb deletion encompassing the entire HNF1B gene) and underwent testing for semaglutide (GLP-1RA).

A female patient, 18 years of age, first arrived at the emergency department at age 12 with symptoms of polyuria, polydipsia, and weight loss ongoing for the past 2 months. Her initial random blood glucose level was found to be 414 mg/dL, leading to a diagnosis of diabetes. She was then put on a regimen of daily doses of long-acting and ultra-short-acting insulin, amounting to 1 unit/kg/day. No insulin-related autoantibodies, including insulin autoantibodies, anti-glutamic acid decarboxylase antibodies, anti-insulin-associated protein two antibodies, or anti-zinc transporter eight antibodies, were found in her blood. Her initial HbA1c level was more than 15%, indicating high blood glucose levels over the past few months, but with no ketosis or ketonuria present and normal blood gases.

Soon after her discharge, her insulin requirements dropped to less than 0.4 units/kg/day due to frequent episodes of low blood sugar. However, her HbA1c levels, indicative of blood glucose control, consistently remained between 6% and 7.7% over the first year. This led to a suspicion of MODY, prompting whole-exome sequencing. The initial results were negative, but a follow-up deletion duplication test revealed that she was a heterozygous carrier of a 1.82-Mb deletion encompassing the entire HNF1B gene (9). This genetic variant was a de novo (parent’s genetic test were negative) and is recognized as a pathogenic confirmation of HNF1B-MODY, a specific subtype that also involves renal cysts and diabetes.

Her family history revealed consanguinity in her healthy parents, with several extended family members suffering from type 2 diabetes but without any kidney dysfunction. During her initial physical examination, she looked healthy and exhibited no unusual physical features such as a goiter or acanthosis nigricans. Her growth parameters were within normal limits; her height of 154 cm fell in the 10th–25th percentile range, her body mass index (BMI) of 26.9 kg/m^2^ fell within the 75th–85th percentile range, and she was at the fifth pubertal stage for breast and pubic hair development.

Laboratory results showed a below-normal magnesium level of 0.5 mmol/L (standard range: 0.86–1.17 mmol/L), while the levels of potassium and other electrolytes were normal. An abdominal ultrasound uncovered a simple cyst on the upper left lobe of the kidney, an unaffected pancreas, and incidental spleen calcification.

**Figure 1:**
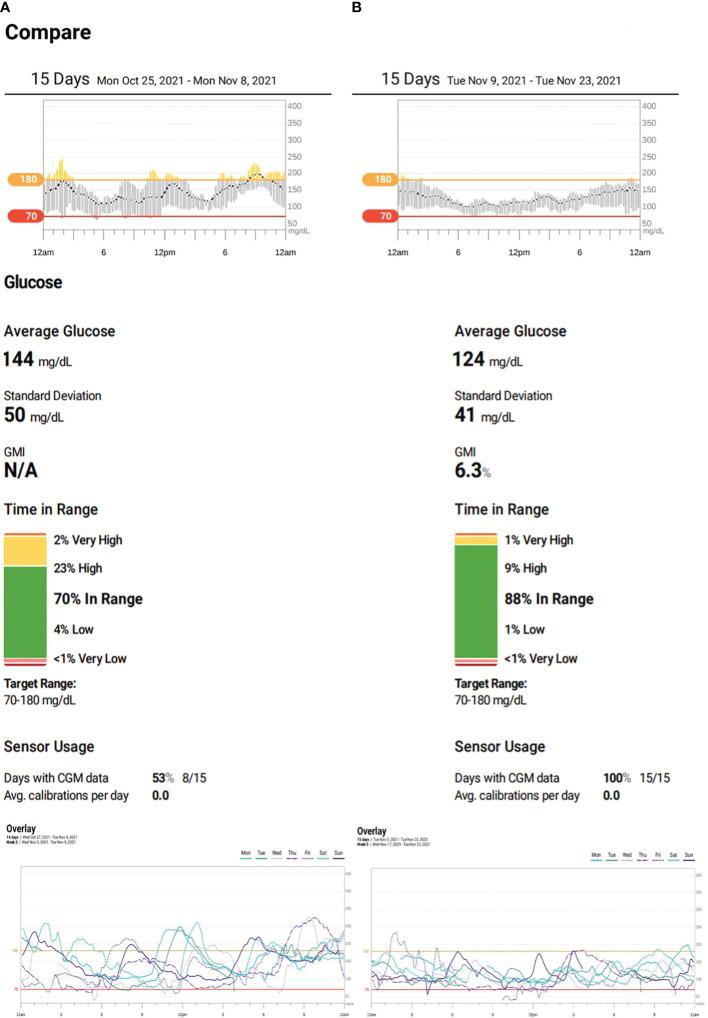
**(A)** Patient’s continuous glucose monitoring (CGM) on insulin therapy. **(B)** Patient’s CGM on semaglutide.

She was treated with magnesium supplements and a daily dose of 0.6–0.7 units/kg/day of both long- and short-acting insulin for several years. After obtaining the necessary ethical approval (IRB/0317/23) and informed consent, an initial dosage of 0.25 mg of semaglutide was administered weekly to the 18-year-old patient for 1 month. This was gradually increased to a maximum dose of 0.5 mg weekly to ensure tolerance. Dexcom Continuous Glucose Monitoring (CGM) was utilized to monitor the patient’s status prior to (**Figure 1A**) and during (**Figure 1B**) the semaglutide trial. This report will present 1 month’s worth of CGM data.

During the trial period, she significantly improved her HbA1c drop from 7.0% to 5.6%—the lowest it has ever been. There were also remarkable improvements in her CGM metrics. Her time-in-range (TIR) went from 70% to 88% and even reached 100% on her best days. Her time-below-range and time-above-range also improved, decreasing from 5% to 1% and 25% to 10%, respectively. The coefficient of variation dropped from 34.3% to 33.4%, and on some days, it reduced further to 27% (**Figures 1A, B**). On top of these, her insulin therapy was discontinued, and her BMI decreased from 24.1 kg/m^2^ (75th–85th percentile) to 20.1 kg/m^2^ (25th–50th percentile).

The semaglutide treatment was halted after 4 months due to various factors. These included side effects like nausea, vomiting, and reduced appetite. Additionally, family issues, exam stress, and a concurrent COVID-19 infection significantly suppressed her appetite. Insulin therapy was resumed as a result. Consequently, her last recorded HbA1c value increased to 7.1% under insulin therapy alone, and her BMI rose to 24.9 kg/m^2^ (**Table 1**).

**Table 1:** BMI, HbA1c, and CGM metric pre-, on, and post-semaglutide treatment.

Parameters	Before semaglutide therapy (insulin 0.7 units/kg/day)	During semaglutide therapy (O.5 mg weekly)	3 months off semaglutide therapy (insulin 0.7 units/kg/day)
BMI kg/m^2^	24.1 (75th-85th percentile)	20.1 (25th-50th percentile)	24.9 (75th-85th percentile)
HbA1c	7%	5.6 %	6.9%
Insulin dose	0.8 u/k/d	Off	0.7 u/k/d
CGM Metric: Average glucose mg/dl TIR TAR1 TAR2 TBR1 TBR2 GMI CV TIR in best days	144 (50 mg/dl SD) 70% 23% 2% 4% <1% 6.8% 34.3% 77%	124 (41 mg/dl SD) 88% 9% 1% 1% <1% 6.3% 33.4% 100%	172 (Via glucometer) - - - - - - -

TIR, time-in-range (glucose level 80–180 mg/dL); TAR1, time-above-range 1 (glucose level 181–250 mg/dL); TAR2, time-above-range 2 (glucose level above 250 mg/dL); TBR1, time-below-range 1 (glucose level below 70 mg/dL); TBR2, time-below-range 2 (glucose level below 54 mg/dL); CV, coefficient of variation.

## Discussion

MODY disrupts insulin biosynthesis and destroys pancreatic beta cells (10), generally manifesting between adolescence and early adulthood (ages 13 to 25) (11, 12). It accounts for approximately 1%–3% of all pediatric and adult diabetes, stemming from monogenic diabetes, a condition mainly impacting beta-cell function due to mutations. The most frequent causes of MODY are mutations in HNF1A and GCK, while HNF4A and HNF1B mutations are less common (13).

The HNF1B gene, which regulates the expression of other genes, binds to specific DNA regions. Its protein is found in numerous organs and tissues, including the liver, lungs, intestines, pancreas, kidneys, reproductive system, and urinary tract. This widespread presence accounts for the disease’s phenotype (14).

HNF1B-MODY can lead to a range of kidney abnormalities, including bilaterally hyperechogenic kidneys visible on prenatal ultrasound, multiple renal cysts, dysplastic multicystic kidneys, single kidneys, horseshoe kidneys, tubular dysfunction, nephropathy caused by hyperuricemia resulting in gout, renal magnesium depletion, and chromophobe renal carcinomas. Comparatively, her kidney symptoms were less severe, presenting as hypomagnesemia and renal cysts.

The pancreas can exhibit extra-renal characteristics such as early-onset diabetes, diabetes that occurs after transplantation, exocrine dysfunction, and the absence of the pancreatic body and tail. In female patients, abnormalities such as a double uterus, underdeveloped vagina, and absence of the uterus have been reported but were not observed in this case. An increased level of liver enzymes can be seen in HNF1B-MODY, but our patient’s level was normal (14). Genetic variants can result from base substitutions leading to different types of mutations, including missense, nonsense, small deletions or insertions, frameshift, and splice mutations. In some instances, like in the present case, a complete gene deletion has been identified (9).

Unlike HNF4A-MODY and HNF1A-MODY, where cell dysfunction predominates, insulin resistance also plays a part in diabetes development in nearly half of HNF1B-MODY mutation carriers. Patients with HNF1B-MODY are often managed with insulin to control their blood glucose because they lack the sulfonylurea sensitivity associated with HNF1A and HNF4A mutations (8).

GLP-1 is a gut-derived incretin hormone that operates through various measures. These include suppressing hepatic gluconeogenesis, amplifying insulin secretion (glucose-dependent), and inhibiting glucagon release (15). Additionally, it curbs appetite, delays gastric emptying, and lessens energy intake (16).

Liraglutide and semaglutide, which stimulate GLP-1 receptors, enhance the efficacy of incretin function and result in lowered fasting and postprandial glucose levels (17). Both medications improve insulin sensitivity, likely due to an overall decrease in body weight (18). These effects were clearly demonstrated by a successful use of daily liraglutide treatment (a GLP-1RA) in a 17-year-old girl with HNF1B-MODY as a main therapy (18). This significant improvement prompted us to consider semaglutide (another GLP-1RA) as another potential weekly treatment option.

The patient underwent treatment for 4 months, initially using a well-tolerated dose of 0.25 mg of semaglutide weekly for 1 month before increasing it to a maximum dose of 0.5 mg. Her glycemic metric, as indicated by her CGM, showed significant improvements. This was due to the GLP-1 agonist, which resulted in reduced hypoglycemia, decreased variability of up to 27% on some days, and a targeted glycemic control of approximately 88%, with a TIR of 100% occasionally. Additionally, her HbA1c reduced to a record low of 5.6% (**Figures 1A, B**; **Table 1**).

Unfortunately, the semaglutide treatment was halted after 4 months despite its effectiveness in enhancing her glycemic metric and establishing tight control through weekly injections. This was due to a variety of reasons, including escalating side effects such as nausea, vomiting, and a reduced appetite. She also had to deal with family problems, examination stress, and a COVID-19 infection, which exacerbated the semaglutide side effects. After discontinuing semaglutide, she reverted to a multiple daily injection regimen of basal-bolus insulin therapy.

This case report deems the response to semaglutide as successful in a short-term trial, as it improved her CGM metrics while she was off insulin therapy. The positive response is likely due to semaglutide’s stimulation of GLP-1 receptors, improving her glycemic control via different mechanisms. These include reducing appetite and weight, enhancing insulin sensitivity, curbing glucagon release, and suppressing hepatic gluconeogenesis. Despite these promising results, we must conduct more extensive studies to assess the long-term safety of this treatment in patients with HNF1B-MODY. Additionally, we should consider testing patients on new GLP-1RA treatments with fewer side effects for future large-scale trials.

The limitation of our study is that it only involves a single HNF1B-MODY patient and spans a short duration.

## Conclusion

This is the premier report demonstrating the effectiveness of once-weekly GLP-1RA (semaglutide) in patients with a complete HNF1B gene deletion (HNF1B-MODY) based on a brief trial. The trial showed the ability to discontinue insulin use and resulted in improved consistency in blood glucose levels, minimized hypoglycemia and hyperglycemia episodes, and a decrease in HbA1c level.

That being said, more extensive studies should be conducted to assess its effect on a larger patient population and its long-term safety for patients with HNF1B-MODY. To safely switch between insulin and other medications, CGM is advised.

## Data availability statement

The original contributions presented in the study are included in the article/supplementary material. Further inquiries can be directed to the corresponding author.

## Ethics statement

The studies involving humans were approved by King Abdullah International Medical Research Center, Riyadh, Saudi Arabia. The studies were conducted in accordance with the local legislation and institutional requirements. The participants provided their written informed consent to participate in this study. Written informed consent was obtained from the individual(s) for the publication of any potentially identifiable images or data included in this article.

## Author contributions

AA: Conceptualization, Investigation, Methodology, Project administration, Supervision, Writing – original draft. BA: Writing – original draft.
